# Improving healthcare consumer effectiveness: An **An**imated, **S**elf-serve, **We**b-based **R**esearch Tool (ANSWER) for people with early rheumatoid arthritis

**DOI:** 10.1186/1472-6947-9-40

**Published:** 2009-08-20

**Authors:** Linda C Li, Paul Adam, Anne F Townsend, Dawn Stacey, Diane Lacaille, Susan Cox, Jessie McGowan, Peter Tugwell, Gerri Sinclair, Kendall Ho, Catherine L Backman

**Affiliations:** 1 Department of Physical Therapy, University of British Columbia, Vancouver, Canada; 2 Arthritis Research Centre of Canada, Vancouver, Canada; 3 Mary Pack Arthritis Program, Vancouver Costal Health, Vancouver, Canada; 4 The W. Maurice Young Centre for Applied Ethics, University of British Columbia, Vancouver, Canada; 5 Faculty of Nursing, University of Ottawa, Ottawa, Canada; 6 Ottawa Health Research Institute, Ottawa, Canada; 7 Division of Rheumatology, Faculty of Medicine, University of British Columbia, Vancouver, Canada; 8 Institute of Population Health, University of Ottawa, Ottawa, Canada; 9 Centre for Digital Media, Vancouver, Canada; 10 Department of Family Medicine, University of British Columbia, Vancouver, Canada; 11 Department of Occupational Science and Occupational Therapy, University of British Columbia, Vancouver, Canada

## Abstract

**Background:**

People with rheumatoid arthritis (RA) should use DMARDs (disease-modifying anti-rheumatic drugs) within the first three months of symptoms in order to prevent irreversible joint damage. However, recent studies report the delay in DMARD use ranges from 6.5 months to 11.5 months in Canada. While most health service delivery interventions are designed to improve the family physician's ability to refer to a rheumatologist and prescribe treatments, relatively little has been done to improve the delivery of credible, relevant, and user-friendly information for individuals to make treatment decisions. To address this care gap, the **An**imated, **S**elf-serve, **We**b-based **R**esearch Tool (ANSWER) will be developed and evaluated to assist people in making decisions about the use of methotrexate, a type of DMARD. The objectives of this project are: 1) to develop ANSWER for people with early RA; and 2) to assess the extent to which ANSWER reduces people's decisional conflict about the use of methotrexate, improves their knowledge about RA, and improves their skills of being 'effective healthcare consumers'.

**Methods/design:**

Consistent with the International Patient Decision Aid Standards, the development process of ANSWER will involve: 1.) creating a storyline and scripts based on the best evidence on the use of methotrexate and other management options in RA, and the contextual factors that affect a patient's decision to use a treatment as found in ERAHSE; 2.) using an interactive design methodology to create, test, analyze and refine the ANSWER prototype; 3.) testing the content and user interface with health professionals and patients; and 4.) conducting a pilot study with 51 patients, who are diagnosed with RA in the past 12 months, to assess the extent to which ANSWER improves the quality of their decisions, knowledge and skills in being effective consumers.

**Discussion:**

We envision that the ANSWER will help accelerate the dissemination of knowledge and skills necessary for people with early RA to make informed choices about treatment and to manage their health. The latest in animation and online technology will ensure ANSWER fills a knowledge translation gap, focusing on the next generation of people living with RA.

## Background

Treatment approaches for RA have changed drastically in the last decade. In the old "pyramid" approach, patients were treated with non-steroidal anti-inflammatory drugs (NSAIDs) without DMARDs until evidence of joint damage appeared. Nowadays, DMARD is used early (i.e., within three months of onset) and consistently, with the aim of eradicating inflammation and preventing joint damage [1-3]. The short-term clinical efficacy of various DMARDs at reducing disease activity, improving physical function and retarding radiographic progression has been demonstrated in RCTs and the subsequent systematic reviews [4-6]. Long-term epidemiological studies have also shown that earlier and more consistent treatment with DMARDs reduces joint destruction and leads to better long-term physical function [7-9]. Delays as brief as three months in starting DMARDs have been associated with poorer long-term outcomes, including greater physical disability [10-12] and joint damage [13-15], and less chance of remission [16]. The evidence suggests that there is a window of opportunity, early in the disease, to successfully control the disease progression; however, only a minority of people with RA used DMARDs after a recent diagnosis [17].

Patients' decisions on medication use can be affected by their concerns about side effects [18]. For example, with methotrexate, a commonly used DMARD, the side effects may include diarrhoea, lung infections, headache, nausea, heartburn, rash, and high liver enzyme, although the occurrence is rare [4,5]. About 10% of patients stopped taking methotrexate due to side effects; however, 9% also stopped the placebo due to the same reasons [4]. Currently, most information on arthritis medications is provided in the written format, which can be challenging especially for people with low literacy. However, while most health service delivery interventions are designed to improve the family physician's ability to refer to a rheumatologist and prescribe medications [19-21], relatively little has been done to improve the delivery of credible, relevant, and user-friendly information for individuals to make treatment decisions.

To address this care gap, we propose to develop and evaluate an **An**imated, **S**elf-serve, **We**b-based **R**esearch Tool (ANSWER) to assist people with a new RA diagnosis to make decisions about using methotrexate. The innovative aspect of ANSWER is its built-in storyline that illustrates common situations that people experience when making decisions about their treatment, and demonstrates the attributes required for effective management of their healthcare. Our specific objectives are to: 1) develop ANSWER for people with early RA; and 2) assess the extent to which ANSWER reduces people's decisional conflict, improves their knowledge about RA, and improves their skills of being 'effective healthcare consumers'.

### Previous research

The development of ANSWER is based on the findings from three projects funded by the Canadian Institutes of Health Research (CIHR; http://webapps.cihr-irsc.gc.ca/funding/Search?p_language=E&p_version=CIHR): 1) the RA Care Gaps project [18]; 2) the ERAHSE (Early RA Help-Seeking Experience) project; and 3) the Effective Consumer project [22].

The RA Care Gaps project surveyed 1,822 patients in British Columbia (BC) and found that the main reasons for not using DMARDs included a lack of physician advice (45%), fear of side effects (26%), preference to avoid medications (24%), and perceived lack of need (23%) [18]. These findings were confirmed by ERAHSE using qualitative interviews of 38 people with newly diagnosed RA (12 months or less). The preliminary data analysis identified common themes about challenges to making treatment decisions or negotiating the healthcare system [23], including: 1) incomplete discussions about benefits and risks of treatment options during medical visits, making it impossible to make an informed choice; 2) frustration with not being heard by the physician, resulting in discontinuation of medical visits by some participants; and 3) frustration with not knowing which, and how to access, health resources even if they knew disease-related information was available.

Another recurring theme identified in ERAHSE was the complex interplay of factors that affected people's treatment decisions. These included the disease characteristics (e.g., insidious versus sudden onset), people's health beliefs, their knowledge and attitudes toward RA and treatments, and their past experience with medications and health professionals. Underlying these factors was the profound feeling of ambivalence that is marked, on one hand, by an aversion to medications because of the anticipated side effects, and on the other, by a fear of the potentially crippling effects of an uncontrolled disease. The circumstance in which people decided to use or not use medications appeared to be influenced by the nature of their symptoms in the context of daily life.

Concerns about side effects were part of the background knowledge that many participants brought to their initial medical consultations. It appeared that these concerns were likely to remain unresolved if they left the doctor's office with unanswered questions or unaddressed concerns about the treatment. This might explain why those who stayed on medications reported having good rapport with their rheumatologists and/or family doctors, and that they were more engaged in communicating and making treatment decisions with their doctors. Interestingly, health education messages, such as those that tout the importance of starting DMARDs within a certain 'window of opportunity' appeared to motivate some ERAHSE participants to begin treatment. These findings highlight the importance of good patient-physician partnerships in people's decisions to use/continue medications, and provide the necessary context for the development of a decision-making tool that mirrors the typical challenges and situations in which people make their treatment choices.

### What are the attributes of an 'effective healthcare consumer'?

Most patient education initiatives aim to empower people with arthritis to become active participants in their healthcare decisions (i.e., 'effective consumers'). The concept of "effective consumer", developed by Tugwell et al. [22] posits that individuals can acquire knowledge and skills that empower them to manage their health and to become more effective users of healthcare resources. Through extensive interviews and collaborations with arthritis patient groups, five essential attributes of effective healthcare consumers were indentified, including the ability to: 1) use health information; 2) clarify personal priorities for disease management; 3) communicate with health professionals; 4) negotiate roles and take control; and 5) make decisions and take action [22,24]. It has been argued that these attributes can be used to guide the development of the next generation of patient education/self-management initiatives [25].

Another major contribution of the effective consumer project was the EC-17 scale [24]. Unlike traditional measures in studies of self-management interventions, which focus on RA outcomes (e.g., pain, joint counts, self-efficacy, life satisfaction, coping, social function) [26], the EC-17 evaluates the person's ability to participate in his/her healthcare. The psychometric properties of the EC-17 are currently being tested in self-management interventions [24]. In the ANSWER project, we will apply the effective consumer attributes to design the knowledge and skills learned by the main character portrayed in the ANSWER storyline, and will use the EC-17 scale in the pilot evaluation.

## Methods/design

ANSWER will be developed based on the rigorous criteria outlined in the International Patient Decision Aid Standards [27,28], and through a collaboration of health researchers, computer animation experts and trainees, and people with RA. Using an iterative design approach, we will create an animated decision aid for users with low health literacy and low-to-average computer skills. Because our main audience is people recently diagnosed with RA, we will focus on creating scenarios that are less threatening to the users (e.g., starting the story in the home and work environment rather than in a hospital). ANSWER will be structured with dual components: stories acted by the main animated character and an interactive decision-making component.

We will adapt Jibaja-Weiss et al.'s Edutainment Decision Aid Model [29], which consists of three inter-linked components: the story; the learning media; and the graphical user interface by which the user interacts with the story and learning media. At different points throughout the story, the user will be required to make decisions. Decision support information (i.e., the risk and benefits of treatment options) will be embedded in both the story and learning media. The animated character will model the skills necessary to be an effective consumer.

The development process of the ANSWER tool involves: 1) writing a brief interactive narrative with multiple storylines for the animated segments and content for the learning segments; 2) refining the storyline and learning segments; and 3) defining the linkages between the story and the learning segments. The storyline will be based on the ERAHSE qualitative interviews. Once the user completes the program, a tailored summary will be generated describing his/her health information, treatment choices, initial decisions made, and concerns about the treatments he/she wishes to discuss with the doctor.

The decision support component will focus on the use of methotrexate. ANSWER will be developed based on the current systematic reviews and clinical practice guidelines about methotrexate [30,31], including the probability for favourable clinical outcomes and side effects. To enable use by computer novices, we will design the graphical user interface of the decision aid such that it resembles a computer game that allows user interaction to enhance the uptake of information. All written content and instructions will be fully narrated.

We will test the content and user interface of the ANSWER prototype with five people from each of the following groups: 1) rheumatologists/arthritis nurses; 2) allied health professionals; 3) people with RA diagnosed ≤ 3 months; 4.) those with RA > 3 months. The time needed to complete the tool will be tracked. After the testing section, participants will be interviewed to obtain comments about the content, presentation and user friendliness. The tool will be refined based on the users' comments.

### Pilot study

A prospective pre-post design will be used in the online pilot study. We will invite people with RA across Canada to participate. Eligible individuals will be those who: 1) are diagnosed with RA; 2) are candidates for methotrexate as indicated by their rheumatologists and have not initiated treatment; and 3) have Internet access. Participants will be recruited from two sources: 1) the Mary Pack Arthritis Program; and 2) patient/consumer groups, including the Canadian Arthritis Patients Alliance and JointHealth. In addition, we will post the information on the Arthritis Research Centre of Canada website. Individuals who are interested may contact the research assistant who will provide details about the study, screen for eligibility and obtain informed consent.

Participants will receive a username and password via e-mail to access the ANSWER testing webpage. They may complete the program at their own pace, and may sign off and return later. At the end of the program, ANSWER will produce a one-page summary for the individual to discuss with his/her rheumatologist/family physician, and then make a decision about the treatment.

### Outcome measures

Participants will be asked to complete the following measures online before and after using the ANSWER: 1) the Decisional Conflict Scale (low literacy version; primary outcome); 2) the EC-17 Scale; and 3) the ACREU (Arthritis Community Research & Evaluation Unit) RA Knowledge Questionnaire. The Decisional Conflict Scale measures personal perceptions of uncertainty in choosing options, factors contributing to uncertainty, and effective decision making [32]. The low literacy version has 10 questions and three response categories. Both internal consistency and test-retest reliability exceed 0.78. In studies of decision aids for non-RA interventions, the effect sizes range form 0.4 to 1.2 [32-34].

The RA Knowledge Questionnaire comprises 31 items covering seven domains: prognosis, coping strategies, pain management, exercise, medication, joint protection, and energy conservation [35]. The questionnaire has demonstrated internal consistency (Cronbach's α = 0.76), test-retest reliability (r = 0.91), and content and construct validity [35]. Also, participants will complete the following measures at baseline: 1.) Health Assessment Questionnaire (HAQ; a validated measure of health status of people with RA); 2.) RADAI (RA Disease Activity Index); and 3.) comorbid conditions and demographic characteristics. The scores of HAQ and RADAI, and the comorbid conditions will be summarized on the one-page summary for participants.

### Sample size and analysis

Based on previous before-and-after studies using the Decisional Conflict Scale, we estimated a difference of 0.3 and a standard deviation of 0.6 [32,33]. This yields a sample size of 43 (α-level = 0.05; 90% power). Assuming that 15% of eligible participants do not complete the task and/or the outcome measures, **51 **participants will be needed. Exploratory analysis using paired t-tests will be done to evaluate the changes in the three outcome measures. Descriptive analysis will be used to summarize participant characteristics and comorbid conditions.

The ANSWER protocol has been approved by the University of British Columbia Behavioural Research Ethics Board (Application number: H09-00898).

### Limitations

Although the selected outcome measures will capture changes in outcomes from the patient's perspective, we will not be able to determine if the ANSWER affects the physician's participation in shared decision-making, or if it improves patient-physician communication during the clinical visit. However, owing to the resources required to collect data from the study participant's physician(s), it is prudent to first demonstrate the value of this new tool for the users in a small pilot study. Favorable results from this study will provide the justification and background data for a full-scale randomized controlled trial that evaluates the effectiveness of ANSWER from the perspectives of both patients and physicians.

## Discussion

This innovative project combines the expertise from six disciplines: health services research (LCL, CLB, DL, DS, PT), clinical research (DL), social science (PA, SC, AFT), information science (JM), knowledge translation (LCL, PT, KH, JM) and digital media (GS), and three stakeholders groups: patients/consumers (Canadian Arthritis Patient Alliance, JointHealth, and Arthritis Research Centre of Canada – Consumer Advisory Board), the clinical community (Mary Pack Arthritis Program), and a non-profit arthritis organization (The Arthritis Society).

A multi-sector, inter-disciplinary team has been assembled to create and evaluate the ANSWER. Guided by Graham's Knowledge-to-Action process [36], this knowledge translation project will directly address one of the recommendations of the *2005 Summit on Standards for Arthritis Prevention and Care *that, "Every Canadian with arthritis must have access to accurate information and education on arthritis that meet a defined set of criteria and are appropriate to their age and stage of disease" [37] We envision that the ANSWER will help accelerate the dissemination of knowledge and skills necessary for people with early RA to make informed choices about treatment and to manage their health.

## Competing interests

The authors declare that they have no competing interests.

## Authors' contributions

The study was conceived by LCL, PA, AT and CLB. All authors contributed to the development of the research protocol. LCL is the principal applicant and PA is the decision-maker co-principal applicant. Writing of the manuscript was led by LCL and all authors approved the final version.

## Pre-publication history

The pre-publication history for this paper can be accessed here:

http://www.biomedcentral.com/1472-6947/9/40/prepub

## References

[B1] Pincus T O'Dell J Combination therapy with multiple disease-modifying antirheumatic drugs in rheumatoid arthritis: A preventive strategy Ann Intern Med 1999 131 768 10577301 10.7326/0003-4819-131-10-199911160-00009

[B2] Fries JF Current treatment paradigms in rheumatoid arthritis Rheumatology 2000 39 Suppl 1 30 35 11001377 10.1093/oxfordjournals.rheumatology.a031492

[B3] Wollheim FA Approaches to rheumatoid arthritis in 2000 Curr Opin Rheumatol 2001 13 193 201 10.1097/00002281-200105000-00008 11333348

[B4] Suarez-Almazor ME Belseck E Shea BJ Tugwell P Wells G Methotrexate for treating rheumatoid arthritis Cochrane Database of Systematic Reviews 3 2008 http://www.cochrane.org/reviews/en/ab000957.html 10.1002/14651858.CD001157 11034702

[B5] Osiri M Shea B Robinson V Suarez-Almazor M Strand V Tugwell P Wells G Leflunomide for treating rheumatoid arthritis Cochrane Database of Systematic Reviews 3 2008 CD002047 10.1002/14651858.CD002047 PMC8437750 12535423

[B6] Suarez-Almazor M Osiri M Emery P Ottawa Methods Group Tugwell P, Shea B, Boers M, Brooks P, Simon LS, Strand V, Wells G Rheumatoid arthritis Evidence-based Rheumatology 2003 London: BMJ Books

[B7] Sokka T Disease-modifying anti-rheumatic drug use according to the 'sawtooth' treatment strategy improves the functional outcome in rheumatoid arthritis: results of a long-term follow-up study with review of the literature Rheumatology 2000 39 34 10.1093/rheumatology/39.1.34 10662871

[B8] Fries JF Williams CA Morfeld D Singh G Sibley J Reduction in long-term disability in patients with rheumatoid arthritis by disease-modifying antirheumatic drug-based treatment strategies Arthritis and Rheumatism 1996 39 616 622 10.1002/art.1780390412 8630111

[B9] Abu-Shakra M Toker R Flusser D Flusser G Friger M Sukenik S Buskila D Clinical and radiographic outcomes of rheumatoid arthritis patients not treated with disease-modifying-drugs Arthritis and Rheumatism 1998 41 1190 1195 10.1002/1529-0131(199807)41:7<1190::AID-ART7>3.0.CO;2-B 9663474

[B10] Munro R Hampson R McEntegart A Thomson EA Madhok R Capell HA Improved functional outcome in patients with early rheumatoid arthritis treated with intramuscular gold: results of a five year prospective study Ann Rheum Dis 1998 57 88 93 10.1136/ard.57.2.88 9613337 PMC1752531

[B11] Tsakonas E Fitzgerald AA Fitzcharles MA Cividino A Thorne JC M'Seffar A Joseph L Bombardier C Esdaile JM Consequences of delayed therapy with second-line agents in rheumatoid arthritis: a 3 year followup on the Hydroxychloroquine in Early Rheumatoid Arthritis (HERA) Study The Journal of Rheumatology 2000 27 623 629 10743799

[B12] Heide A van der Jacobs JW Bijlsma JW Hcurkens AH Booma-Frankfort C Veen MJ van der Haanen HC Hofman DM The effectiveness of early treatment with second-line antirheumatic drugs. A Randomized, controlled trial Ann Intern Med 1996 124 699 707 8633829 10.7326/0003-4819-124-8-199604150-00001

[B13] Lard LR Visser H Speyer I Horst-Bruinsma IE vander Zwinderman AH Breedveld FC Hazes JMW Early versus delayed treatment in patients with recent-onset rheumatoid arthritis: comparison of two cohorts who received different treatment strategies The American Journal of Medicine 2001 111 446 451 10.1016/S0002-9343(01)00872-5 11690569

[B14] Buckland-Wright J Quantitative microfocal radiography detects changes in erosion area in patients with early RA treated with myochrisine The Journal of Rheumatology 1993 20 243 247 8474059

[B15] Egsmose C Lund B Borg G Pettersson H Berg E Brodin U Trang L Patients with rheumatoid arthritis benefit from early 2nd line therapy: 5 year followup of a prospective double blind placebo controlled study The Journal of Rheumatology 1995 22 2208 2213 8835550

[B16] Mottonen T Hannonen P Korpela M Nissila M Kautiainen H Honen J Laasonen L Kaipiainen-Seppanen O Franzen P Helve T FINnish Rheumatoid Arthritis Combination therapy Arthritis and Rheumatism 2002 46 894 898 10.1002/art.10135 11953964

[B17] Lacaille D Anis AH Guh DP Esdaile JM Gaps in care for rheumatoid arthritis: a population study Arthritis Rheum 2005 53 241 248 10.1002/art.21077 15818655

[B18] Lacaille D Rogers P Why are people with rheumatoid arthritis not using DMARDs? Understanding gaps in care Arthritis Rheum 2007 56 S86

[B19] Glazier RH Badley EM Lineker SC Wilkins AL Bell MJ Getting a Grip on Arthritis: an educational intervention for the diagnosis and treatment of arthritis in primary care J Rheumatol 2005 32 137 142 15630739

[B20] Schulpen GJ Vierhout WP Heijde DM van der Landewe RB Winkens RA van Der LS Joint consultation of general practitioner and rheumatologist: does it matter Ann Rheum Dis 2003 62 159 161 10.1136/ard.62.2.159 12525386 PMC1754440

[B21] Gormley GJ Steele WK Gilliland A Leggett P Wright GD Bell AL Matthews C Meenagh G Wylie E Mulligan R Can diagnostic triage by general practitioners or rheumatology nurses improve the positive predictive value of referrals to early arthritis clinics? Rheumatology 2003 42 763 768 10.1093/rheumatology/keg213 12730536

[B22] Tugwell PS Wilson AJ Brooks PM Driedger SM Gallois C O'Connor AM Qualman A Santesso N Wale J Wells GA Attributes and skills of an effective musculoskeletal consumer J Rheumatol 2005 32 2257 2261 16265713

[B23] Townsend A Adam PM Cox SM Li L Help-seeking in early rheumatoid arthritis: From onset to early-post diagnosis (Abstract 1259) Arthritis Rheum 2007 56 S507

[B24] Kristjansson E Tugwell PS Wilson AJ Brooks PM Driedger SM Gallois C O'Connor AM Qualman A Santesso N Wale J Development of the effective musculoskeletal consumer scale J Rheumatol 2007 34 1392 1400 17552066

[B25] Tugwell PS Santesso NA O'Connor AM Wilson AJ Effective Consumer Investigative Group Knowledge translation for effective consumers Physical Therapy 2007 87 1728 1738 17906288 10.2522/ptj.20070056

[B26] Newman S Steed L Mulligan K Self-management interventions for chronic illness Lancet 2004 364 1523 1537 10.1016/S0140-6736(04)17277-2 15500899

[B27] International Patient Decision Aid Standards (IPDAS) checklist 2008 Ottawa Health Research Institute http://decisionaid.ohri.ca/methods.html#checklist

[B28] Elwyn G O'Connor A Stacey D Volk R Edwards A Coulter A Thomson R Barratt A Barry M Bernstein S Developing a quality criteria framework for patient decision aids: online international Delphi consensus process BMJ 2006 333 417 10.1136/bmj.38926.629329.AE 16908462 PMC1553508

[B29] Jibaja-Weiss ML Entertainment education for informed breast cancer treatment decisions in low-literate women: development and initial evaluation of a patient decision aid Journal of Cancer Education 2006 21 133 139 10.1207/s15430154jce2103_8 17371175

[B30] Visser K Heijde D van der Optimal dosage and route of administration of methotrexate in rheumatoid arthritis: a systematic review of the literature Ann Rheum Dis 2009 68 1094 1099 10.1136/ard.2008.092668 19033290 PMC2689521

[B31] Visser K Katchamart W Loza E Martinez-Lopez JA Salliot C Trudeau J Bombardier C Carmona L Heijde D van der Bijlsma JW Multinational evidence-based recommendations for the use of methotrexate in rheumatic disorders with a focus on rheumatoid arthritis: integrating systematic literature research and expert opinion of a broad international panel of rheumatologists in the 3E Initiative Ann Rheum Dis 2009 68 1086 1693 10.1136/ard.2008.094474 19033291 PMC2689523

[B32] O'Connor A User Manual – Decisional Conflict Scale 2005 Ottawa, Ottawa Health Research Institute http://decisionaid.ohri.ca/docs/develop/User_Manuals/UM_Decisional_Conflict.pdf

[B33] O'Connor AM Tugwell P Wells GA Elmslie T Jolly E Hollingworth G McPherson R Bunn H Graham I Drake E A decision aid for women considering hormone therapy after menopause: decision support framework and evaluation Patient Education and Counseling 1998 33 267 279 10.1016/S0738-3991(98)00026-3 9731164

[B34] Drake ER Engler-Todd L O'Connor AM Surh LC Alasdair H Development and Evaluation of a Decision Aid About Prenatal Testing for Women of Advanced Maternal Age Author Journal of Genetic Counseling 1999 8 217 233 10.1023/A:1022998415890 26142262

[B35] Lineker SC Badley EM Hughes EA Bell MJ Development of an instrument to measure knowledge in individuals with rheumatoid arthritis: the ACREU rheumatoid arthritis knowledge questionnaire J Rheumatol 1997 24 647 653 9101496

[B36] Graham ID Logan J Harrison M Straus S Tetroe J Caswell W Robinson N Lost in Knowledge Translation: Time for a Map? Journal of Continuing Education in the Health Professions 2006 26 13 24 10.1002/chp.47 16557505

[B37] Alliance for the Canadian Arthritis Program Arthritis isn't a big deal until you get it. Ask 4 million Canadians. Report from the Summit on Standards for Arthritis Prevention and Care, November 1–2, 2005, Ottawa, Ontario, Canada 2006 Toronto, ON: Alliance for the Canadian Arthritis Program

